# Increased Skin Tumor Incidence and Keratinocyte Hyper-Proliferation in a Mouse Model of Down Syndrome

**DOI:** 10.1371/journal.pone.0146570

**Published:** 2016-01-11

**Authors:** Annan Yang, Duane Currier, Jennifer L. Poitras, Roger H. Reeves

**Affiliations:** 1 Department of Physiology and McKusick Nathans Institute for Genetic Medicine, Johns Hopkins University School of Medicine, Baltimore, Maryland, United States of America; 2 Department of Oncology, Johns Hopkins University School of Medicine, Baltimore, Maryland, United States of America; University of Connecticut Health Center, UNITED STATES

## Abstract

Down syndrome (DS) is a genetic disorder caused by the presence of an extra copy of human chromosome 21 (Hsa21). People with DS display multiple clinical traits as a result of the dosage imbalance of several hundred genes. While many outcomes of trisomy are deleterious, epidemiological studies have shown a significant risk reduction for most solid tumors in DS. Reduced tumor incidence has also been demonstrated in functional studies using trisomic DS mouse models. Therefore, it was interesting to find that Ts1Rhr trisomic mice developed more papillomas than did their euploid littermates in a DMBA-TPA chemical carcinogenesis paradigm. Papillomas in Ts1Rhr mice also proliferated faster. The increased proliferation was likely caused by a stronger response of trisomy to TPA induction. Treatment with TPA caused hyperkeratosis to a greater degree in Ts1Rhr mice than in euploid, reminiscent of hyperkeratosis seen in people with DS. Cultured trisomic keratinocytes also showed increased TPA-induced proliferation compared to euploid controls. These outcomes suggest that altered gene expression in trisomy could elevate a proliferation signalling pathway. Gene expression analysis of cultured keratinocytes revealed upregulation of several trisomic and disomic genes may contribute to this hyperproliferation. The contributions of these genes to hyper-proliferation were further validated in a siRNA knockdown experiment. The unexpected findings reported here add a new aspect to our understanding of tumorigenesis with clinical implications for DS and demonstrates the complexity of the tumor repression phenotype in this frequent condition.

## Introduction

Down syndrome (DS) results from the inheritance of three copies of human chromosome 21 (Hsa21). Epidemiological studies spanning 50 years report conflicting results regarding the relative risk of tumor development in the DS population. However, the preponderance of recent studies and biological experiments conducted in trisomic mouse models support the reduction of many types of tumors on a trisomic background and implicate several candidate genes and mechanisms for tumor repression [1, 2]. There does not appear to be a universal mechanism wherein over-expression of one or a few trisomic genes could explain the reduced incidence of many types of cancers in individuals who have DS. Extrapolation of the basis for protection from solid tumors by trisomy could form the basis for cancer prophylaxis in the larger population.

The overexpression of hundreds of genes in Down syndrome disrupts many signalling pathways, including oncogenic and tumour suppressive pathways. Mouse models with different trisomic segments orthologous genes to Hsa21 (or carrying the human chromosome itself) have been used to identify gene(s) that may contribute to cancer resistance phenotypes reflecting those seen in people with DS [3, 4]. Ts65Dn and Ts1Rhr are genetic mouse models of DS that are trisomic, respectively, for ca. 100 and 32 genes from mouse chromosome 16 (Mmu16) that are orthologs of genes on human chromosome 21 (Hsa21) [5, 6]. Both models show significant repression of intestinal adenomas in *Apc*^*Min*^ mice, and this is strongly correlated with dosage of the *Ets2* gene, one of the 32 genes that is trisomic in Ts1Rhr [3]. “Subtracting” one copy of *Ets2* from the three copies in Ts1Rhr, *Apc*^*Min*^ mice results in significantly increased tumor number relative to Ts1Rhr; *Ets2*^*+/-*^ mice with only one functional copy have a dramatic increase in tumor number. The Ts65Dn background also reduces sarcoma incidence and extends survival significantly in NP-cis mice, which develop sarcomas, carcinomas and lymphomas [4]. Further, *Rcan1* decreased tumor growth in the xenografts into a trisomic background due to effects on angiogenesis, proliferation and/or apoptosis depending on the tumor cell lines used for transplant [7–9]. Together, the epidemiological studies in people and the demonstration of strong biological effects in mouse models confirm that tumor incidence for multiple cancer types is repressed by trisomic gene dosage effects.

Three studies have reported a reduced incidence of various skin cancers in people with DS with limited samples [10, 11]. To study skin cancer development in a DS mouse model, we used the two-stage DMBA-TPA carcinogenesis assay [12]. The initiation step involves generation of irreversible *Hras* mutations by DMBA. Cell proliferation promoted by TPA allowed accumulation of further somatic mutations, leading to the irreversible malignant conversion of benign tumors, such as activation of protein kinase C (PKC) [13]. In the Ts65Dn hippocampus, altered PKC activity was found and may be responsible for the impaired hippocampal synaptic plasticity [14]. A few genes in the Down syndrome region were found to affect PKC pathway, such as overexpression of PFKL increase PKC level in PC12 cells [15].

Here we used Ts1Rhr to examine the effects of trisomy on the incidence of skin tumors in a carcinogen-induced skin cancer model. Then we used in vitro keratinocyte culture and gene expression profiling to probe genes overexpressed in Ts1Rhr that could affect proliferation. We validated a role for several critical genes, trisomic in Ts1Rhr, that contributed to increased proliferation by RNAi screening.

## Materials and Methods

### Chemicals

DMBA (D3254) and TPA were obtained from Sigma-Aldrich, St. Louis, MO. Acetone was purchased from J.T. Baker, Phillipsburg, NJ.

### Mice

All procedures in this study were carried out in strict accordance with the recommendations in the Guide for the Care and Use of Laboratory Animals of the National Institutes of Health and approved by the Johns Hopkins Animal Care and Use Committee (Protocol number: MO12M427). Animals were monitored twice each day and no unexpected deaths were observed. Animals exhibiting signs of distress were examined by veterinarian and given 4–5 mg/kg carprofen subcutaneously. Animals with the following conditions such as inactive, hunched and poorly groomed were examined by a veterinarian who decided if euthanasia was appropriate. Animals were euthanized with CO_2_ inhalation followed by cervical dislocation. Ts1Rhr mice on the C57BL/6J genetic background from our colony were backcrossed onto FVB/N (The Jackson Laboratory, Bar Harbor) for at least 6 generations. For all in vivo experiments, the dorsal skin was shaved 7 days prior to treatment. For the two-stage carcinogenesis experiment, 8–12 week old mice were treated with a single application of DMBA (400 nmol in 100 μl acetone) followed by twice-weekly application of TPA (5 nmol in 100μl acetone) for 18 weeks [16]. Only tumors that persisted for more than two weeks were recorded. Mice were examined weekly for tumor presence and the size of each tumor was measured by calliper and recorded. At the end of study, we had full record of the number of tumor as well as tumor size for each mouse on weekly basis. The end point of survival was determined either when the largest tumor reached the upper limit of 1.5 cm in diameter or when an animal showed signs of distress, as approved by The Johns Hopkins Animal Care and Use Committee. For tumor incidence, we plot average tumor number of each group along time. For tumor size, we first took average of all tumor on one mouse and then took average tumor size of each group then plot it along the time.

### Epidermal thickness measurement

The dorsal skin of 5 week old mice was shaved. One week later, TPA (5 nmol in 100 μl acetone) was applied once a day for three days and mice were sacrificed two days later. The dorsal skin was prepared for histology so as to minimize variation due to cutting angle and measurements were always made in the flat area between hair follicles to avoid epidermal pegs. All samples were processed blind to genotype. To further reduce the variance caused by region selection bias, we scanned every slide and picked the three thickest regions to photograph and three measurements were made in every picture. Epidermal thickness was measured using NIS-Elements BR3.0 software.

### Keratinocyte cultures and in vitro treatment

Skin keratinocytes were isolated from P1 pups and cultured as described [17]. For flow cytometry, aliquots of 10^6^ cells were collected and resuspended in 1 ml of hypotonic DNA staining buffer (0.1% Sodium citrate, 0.3% Triton–x 100, 0.01% Propidium iodide and 0.02g/l of Ribonuclease A in distilled water). Samples were held at 4°C protected from light for 30 min before acquisition on the flow cytometer (FACScalibur, BD bioscience, Franklin Lakes). For siRNA transfected samples, all procedures were done 24 hours after transfection. Cells plated in one well of a 6-well plate for each genotype were treated with 0.2μM TPA for 12 h, exposed to 50 μM EdU for 2 h, fixed, and processed for EdU staining (Click-iT EdU Imaging Kits, Invitrogen, Carlsbad). Each keratinocyte line was derived from one pup and individually genotyped. All experiments were at least repeated three times with biological replicates in each genotype group.

### RNA extraction and RT-PCR

Total RNA from skin tumors or cells was extracted using Trizol (Invitrogen, Carlsbad) or RNeasy Mini Kits (Qiagen, Venlo). DNA was removed from RNA with using DNA-free (Invitrogen, Carlsbad). Total RNA was reverse-transcribed by GoScript Reverse Transcription System (Promega, Madison). All Probes for Taqman assays are given in S1 Table (Applied Biosystem, CA). Sequences for SYBRprimers were from PrimerBank [18] or qPrimerDepot [19]. Efficiency tests were done for gene primers. A melting curve was done for all SYBR primers to confirm that there was a single product.

### Microarray and Differential Expression Analysis

All RNA samples were verified for integrity using a Bioanalyzer (Agilent, Santa Clara). The Lowe Family Genomics Core at Johns Hopkins University hybridized total RNA samples to MouseRef-8 v2.0 Expression BeadChip (Illumina, San Diego) and performed quality control analysis. All differential expression analysis was carried out using the statistical computing environment, R, with the limma [20–22] and vsn [23] packages installed. Raw signal intensities were normalized using the Variance Stabilizing Transformation method. Probes were removed from further analysis if less than 4 samples of one genotype had detection p-values greater than 0.01 (1 percent of the 1000 negative control probes had a higher signal intensity). Differentially expressed genes were identified using a standard limma workflow incorporating paired samples into the linear models. Briefly, to identify differentially expressed probes between treated and untreated samples, data for each genotype was analyzed independently using a design matrix that included the source of the cells as well as treatment. To identify probes that were differentially expressed between genotypes in the untreated condition, a design matrix was constructed that included only genotype and treatment. Filtered, normalized expression data was fitted to linear models based on the design matrices. Contrasts between the fitted models of the groups of interest were made and the probes were ranked using an empirical Bayesian method [20]. The expression data reported in this paper have been deposited in the Gene Expression Omnibus (GEO) (http://www.ncbi.nlm.nih.gov/geo) database (series accession number GSE40382).

### siRNA knockdown assay

On-target plus siRNAs (0.2 nMol) were ordered from Dharmacon (Pittsburgh, PA) (S2 Table) and resuspended according to manufacturer’s instruction. Each on-target plus siRNA contains four different siRNAs for the target gene. To knock down gene expression, 50nM siRNA and 0.25 μl Dharmafect 1 were used for each well in a 96-well plate. Triplicate knockdowns were performed and four fields in each well were counted to measure EdU+ cell percentage.

### Statistical analysis

Data were reported as mean ± s.d. unless specifically mentioned. P values were calculated using Student’s t-test (Excel) while the log-rank test and ANOVA were computed using R with the survival package [24].

## Results

### Ts1Rhr has elevated tumor burden and decreased survival

To test possible protective effects of trisomy, we inbred the trisomic segment of Ts1Rhr [5] onto the FVB/N strain to increase susceptibility to the effects of TPA and DMBA. We administered DMBA-TPA treatment to produce two comparing groups of mice: euploid (WT) and FVB.Ts1Rhr (Ts). Carcinogen treatment was administered to all mice at the same stage of skin development during the telogen phase of hair-follicle cycling (8–12 wks old) as susceptibility may vary at different phase of hair growth [25].

**Fig 1:**
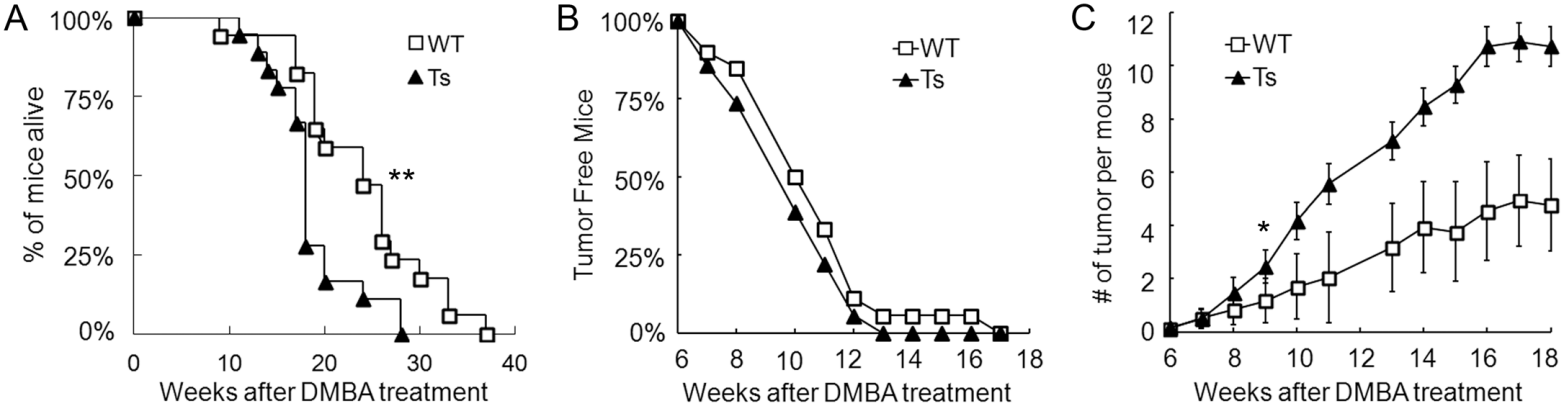
Survival, tumor initiation, and number in DMBA-TPA treated mice. Tumor number and size were measured twice a week from the initial DMBA treatment for 20 weeks. (A) WT (n = 20) survived significantly longer than Ts1Rhr (n = 21) mice. **p = 0.007 by Log-rank test. (B) The tumor initiation plotted versus time (weeks) for WT and Ts1Rhr mice. (C) Tumor number per mouse plotted versus time was not different in Ts and WT mice (error bar is SE). From week 9 on, Ts1Rhr developed significantly more tumors per individual compared to WT (ANOVA, *p<0.04).

In contrast to our expectation based on previous experiments showing reduced tumor incidence and progression in trisomic mice, tumors in Ts mice in this experiment had significantly reduced survival compared to WT littermates (Chisq = 7.2 on 1 degree of freedom, p = 0.007 by Log-rank test) (Fig 1A). Very few mice exhibited distress and thus, most were sacrificed when the largest tumor reached the upper limit of 1.5 cm (see Methods). The median survival was 121 days for Ts versus 167 days for WT groups. We then investigated several properties of growth and transformation. We saw no difference between WT and Ts groups in the timing of tumor initiation; no mouse developed tumors before week 7 and 100% of mice developed tumors by week 12–16 after DMBA treatment for both groups (Fig 1B). However, comparison of tumor burden between the two groups showed that Ts mice developed significantly more tumors than WT mice from 9 weeks post-DMBA (Fig 1C) (ANOVA, p<0.04). The increased tumor incidence in Ts mice persisted through the 18 weeks of the experiment. The ratio of papilloma versus squamous cell carcinoma (SCC) was also not different between the two groups (S1 Fig). Due to relatively early termination of the experiment based on the tumor size restriction, we did not observe any metastasis in our model. In summary, the reduced survival of trisomic mice is correlated with the increased tumor burden compared to WT groups.

### Increased tumor growth and hyperkeratosis in Ts1Rhr induced by TPA *in vivo* and *in vitro*

**Fig 2:**
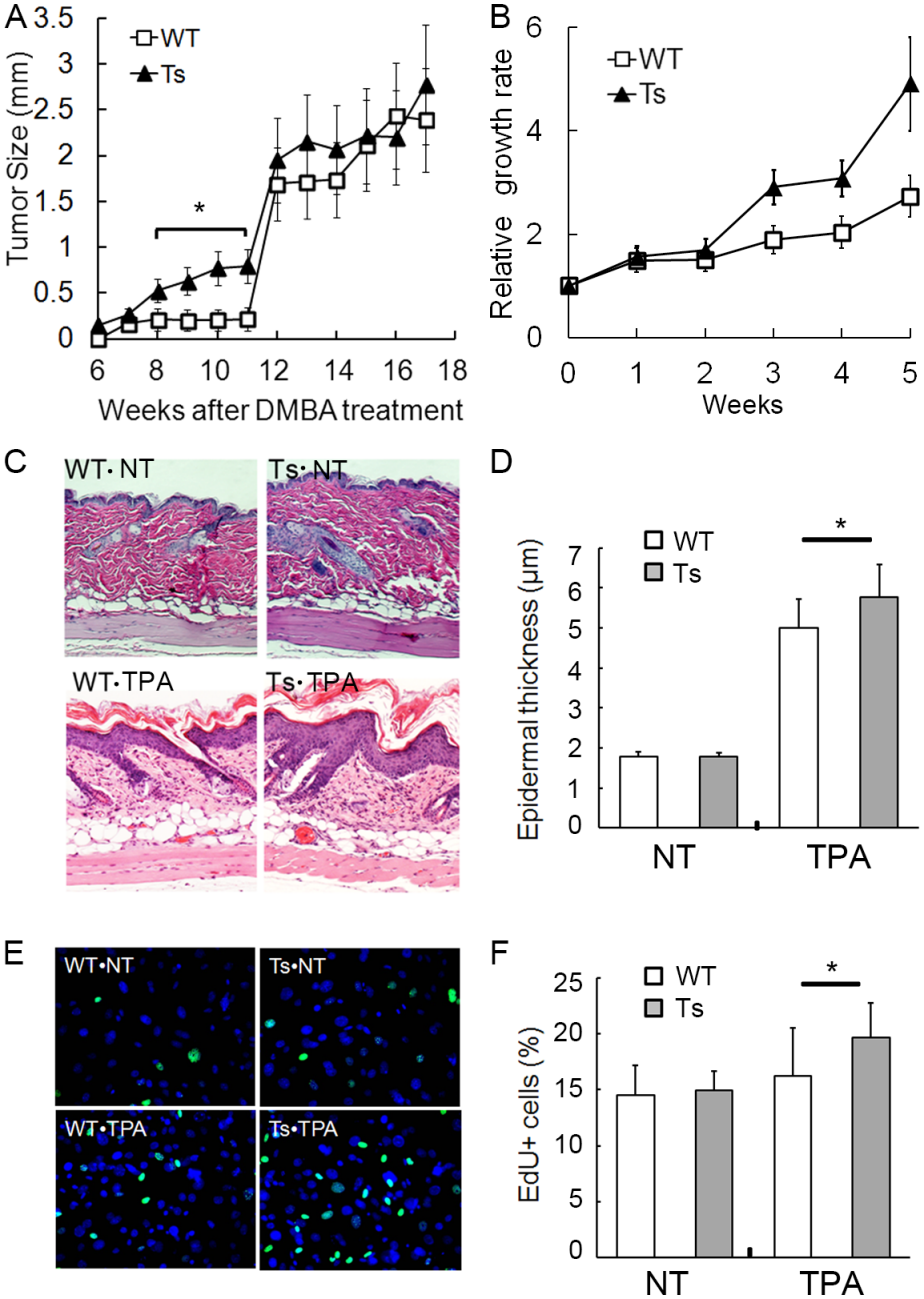
Ts1Rhr mice demonstrate increased proliferation and TPA-induced hyperkeratosis *in vivo* and *in vitro*. (A) The average tumor size (mm) ± S.E. was plotted versus time in WT and Ts groups. * T-test, p<0.05. (B) Tumors showed increased relative growth rate in Ts compared to WT (*p<0.028). (C) H&E stain of WT and Ts skin without TPA treatment (WT-NT and Ts-NT). H&E stain of TPA-treated skin (WT-TPA and Ts-TPA). (D) There is no difference in thickness of the epidermis between WT (n = 12) and Ts (n = 14) without TPA treatment (NT group). The epidermis of Ts (n = 14) is significantly thicker than that of WT (n = 16) (*P = 0.023, T-test). (E) EdU staining of keratinocytes +/- TPA treatment. (F) Quantitation of cells in (2E) showed significantly more EdU positive cells in Ts cultures following TPA treatment (*p = 0.043, T-test). NT, no TPA treatment; TPA, treatment with TPA (see Methods).

Next we compared tumor growth rate group-wise as well as individually. Comparing average tumor size of WT and Ts, there was a significant increase of growth in Ts group from week 8 to 11 (T-test *p<0.05) (Fig 2A). After week 12 there was not a statistically significant difference in average tumor size between WT and Ts, partly due to that more newly emerged (smaller) papillomas in Ts mice tend to decrease overall average size. Accordingly, we selected 10 individual tumors each in the WT and Ts groups and plotted the relative growth rate over time (Fig 2B). This analysis showed that Ts tumors grew significantly faster than did WT. We then examined the effect of TPA alone on Ts and WT skin without DMBA treatment. There was no difference in average epidermal thickness in untreated mice (1.78±0.12 μm and 1.78±0.11 μm in WT and Ts, respectively) (Fig 2C and 2D). However, topical application of TPA for three consecutive days produced significantly more epidermal thickening in Ts mice. Average epidermal thickness increased to 5.0±0.7 μm in WT and 5.8±0.8 μm in Ts (p = 0.023) (Fig 2C and 2D).

The increased responsiveness of trisomic skin to TPA was reflected in isolated keratinocyte cultures (Figures A and B in S1 File). Ts keratinocytes harvested from P1 mice showed increased incorporation of EdU label in culture after TPA treatment compared to WT (Fig 2E and 2F). There was no difference in the number of cycling cells before TPA treatment (Figures A and C in S2 File), a significantly higher percentage of Ts keratinocytes entered S+G2/M phase in response to TPA compared to WT (5.5 vs. 3.2%, *p = 0.034) (Figures B and D in S2 File). This suggests that the trisomic population contained a higher proportion of TPA-responsive cells or that they cycle substantially faster. Thus the in vitro culture findings mirror the in vivo proliferative responses of trisomic and euploid skin to TPA.

### Gene expression profile changes of keratinocytes induced by TPA treatment

**Fig 3:**
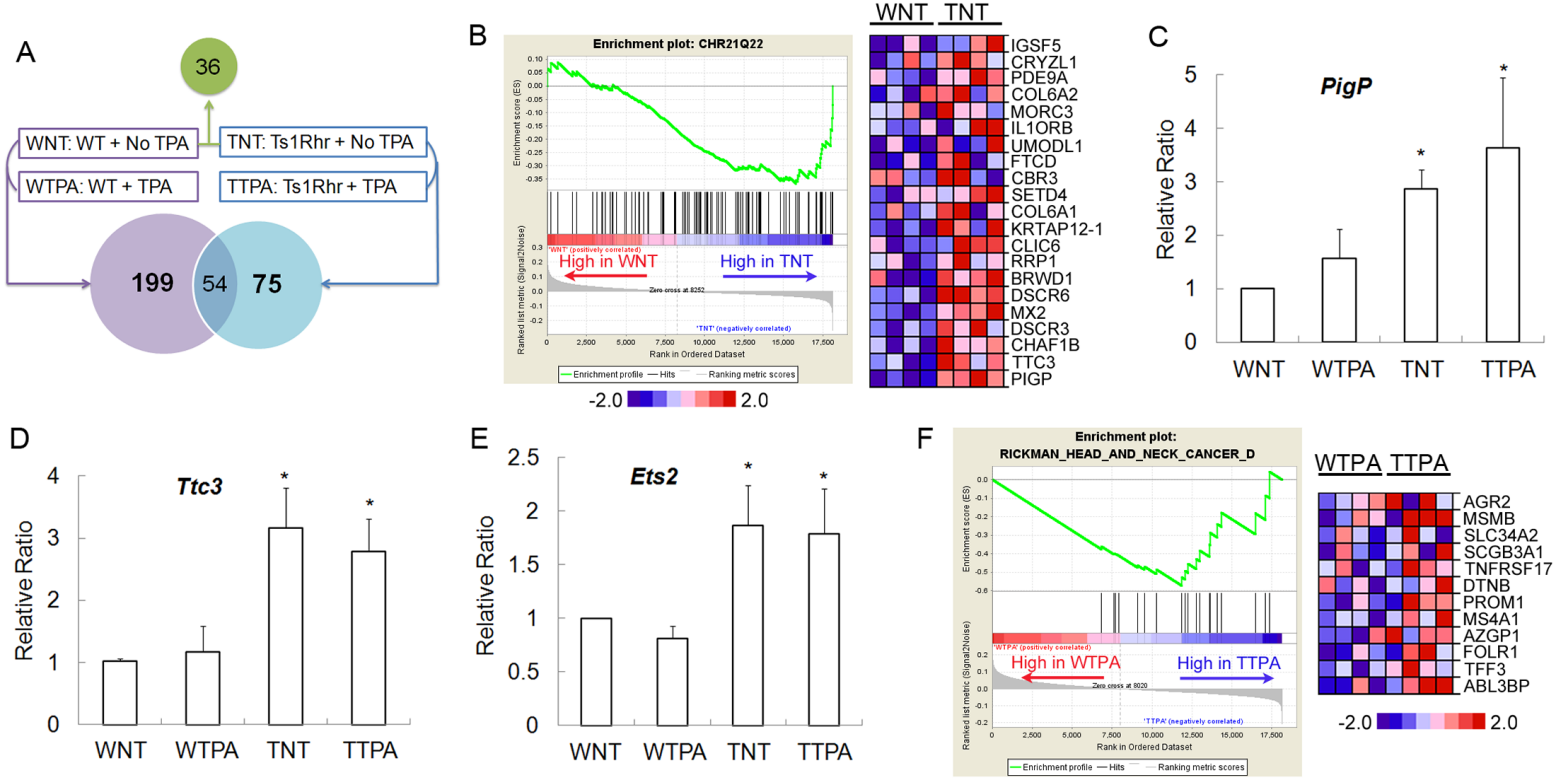
Microarray analysis and RT-PCR validation of gene expression in keratinocytes. (A) TPA treated and untreated keratinocytes isolated from trisomic (Ts) and euploid (WT) mice are compared in this figure (n = 4 in each group). Differentially expressed genes are depicted in the Venn diagram. (B) Gene Set Enrichment Analysis (GSEA) was used to identify gene sets that exhibited significant overlaps with those gene differentially expressed between WT-NT and Ts-NT. Enrichment plot (left panel) and Heat map (right panel) for the Chr21q22 region gene set from GSEA analysis of WT-NT vs. Ts-NT keratinocytes. The relative ratios of transcripts are shown for trisomic genes. (C) *PigP*, (D) *Ttc3* and (E) *Ets2* in WT and Ts keratinocyte cultures before and after TPA treatment (*p<0.01). (F) Gene enrichment analysis found genes in the signature of head and neck squamous cell carcinoma significantly upregulated in the Ts-TPA group vs. WT-TPA (left panel). A heat map shows core genes upregulated in the Ts-TPA group (right panel).

We used microarray analysis to examine RNA expression levels in resting and TPA-treated keratinocytes from WT and Ts mice (Fig 3A). The array contained 25,697 probes of which 8,794 had intensities greater than the detection threshold (see Methods). Of these, 36 genes showed a significant difference in expression between WT and Ts resting keratinocytes (WT-NT and Ts-NT, respectively). Gene enrichment analysis comparing WT-NT and Ts-NT samples indicated a significant collective upregulation of genes corresponding to the Chr21q22 region in human beings (CHR21Q22 gene set, p < 0.05) (Fig 3B) [26, 27]. This is the region where orthologs of genes triplicated in Ts1Rhr are found, reflecting the expected gene dosage effect. Among the 32 genes that are trisomic in Ts1Rhr mice, 17 were below the detection threshold. When the remaining genes triplicated in Ts1Rhr were considered individually, only *PigP* was significantly overexpressed in TS-NT keratinocytes compared to WT-NT [5]. However, several trisomic genes were significantly upregulated when analyzed by the more sensitive qPCR technique (Fig 3C–3E; Table 1, “Ts” column). No other significantly enriched gene sets were seen when comparing genes expressed differentially in WT-NT and TS-NT.

**Table 1:** Relative RNA expression level (qPCR) after siRNA knock-down in Ts keratinocyte (fold change relative to WT).

Target Gene (TG)	Ts	Ts-Scr^a^	Ts-siTG^b^
*PigP*	1.7	2	0.1*
*Ttc3*	2.2	1.2	0.3*
*Ets2*	1.7	1.4	0.8*
*Cbr3*	1.7	2	0.3*
*Chaf1b*	1.4	1.2	0.3*
***Rcan1***^c^	0.9	1.1	0.3*
*Dyrk1A*	1.8	1.6	0.9*
*Kcnj15*	1.4	0.8	0.3*
*Bace2*	1.1	0.9	0.2*
***Elk3***	0.8	0.8	0.4*
***Rab32***	1	1.1	0.1*
***Mmp3***	2.4	2.9	0.5*
***Mmp9***	1.4	1.6	0.2*

* Indicates a significant reduction in gene expression in siRNA treated cells compare to either original cells or si-Scr treated cells (p<0.05).

^a^ Ts keratinocyte treated with scrambled siRNA.

^b^ Ts keratinocyte treated with sets of four target gene siRNAs.

^c^ Genes in bold are disomic in the Ts1Rhr mouse

Pairwise comparison using a normalized p-value of 0.05 as cut off identified 199 genes that were differentially expressed in the WT-no treatment (WT-NT) keratinocyte compared to WT-TPA cells in response to TPA treatment (Fig 3A). In trisomic keratinocytes, 75 genes were differentially expressed between Ts-NT and Ts-TPA. Fifty-four of these genes were altered in both genotypes. Among 21 genes with altered expression only in Ts-TPA-treated cells, *Elk3* [28] and *Rab32* [29] have well known functions in the *Ras* pathway, while *Mmp3* and *Mmp9* are involved in carcinogenesis [30]. Comparing transcript profiles of the WT-TPA and Ts-TPA groups, the gene set for an intrinsic group in head and neck squamous cell carcinoma (HNSCC) [31] was significantly up-regulated in TPA-treated trisomic keratinocytes (normalized p-value<0.001) (Fig 3F).

### Candidate gene contributions to keratinocyte proliferation

We carried out functional experiments with siRNA to determine whether increased expression of any of 13 candidate genes identified by microarray analysis was associated with the hyperproliferative response of Ts cells to TPA (S2 Table). These 13 genes include 8 that are trisomic in Ts1Rhr; *Rcan1*, an Hsa21 ortholog that is not trisomic in Ts1Rhr but has been reported to interact with the trisomic gene, *Dyrk1a*; and 5 disomic candidate genes (Table 1) that were differentially expressed and whose functions suggest possible contributions to the proliferation response (Figure B in S1 File, S2 Table). Each ON-TARGET siRNA mix includes four different siRNAs per target gene to ensure specific targeting. A scrambled siRNA control (ScrRNA) was included in all experiments as control. All target genes showed significantly reduced transcript levels after siRNA knock down (Table 1).

**Fig 4:**
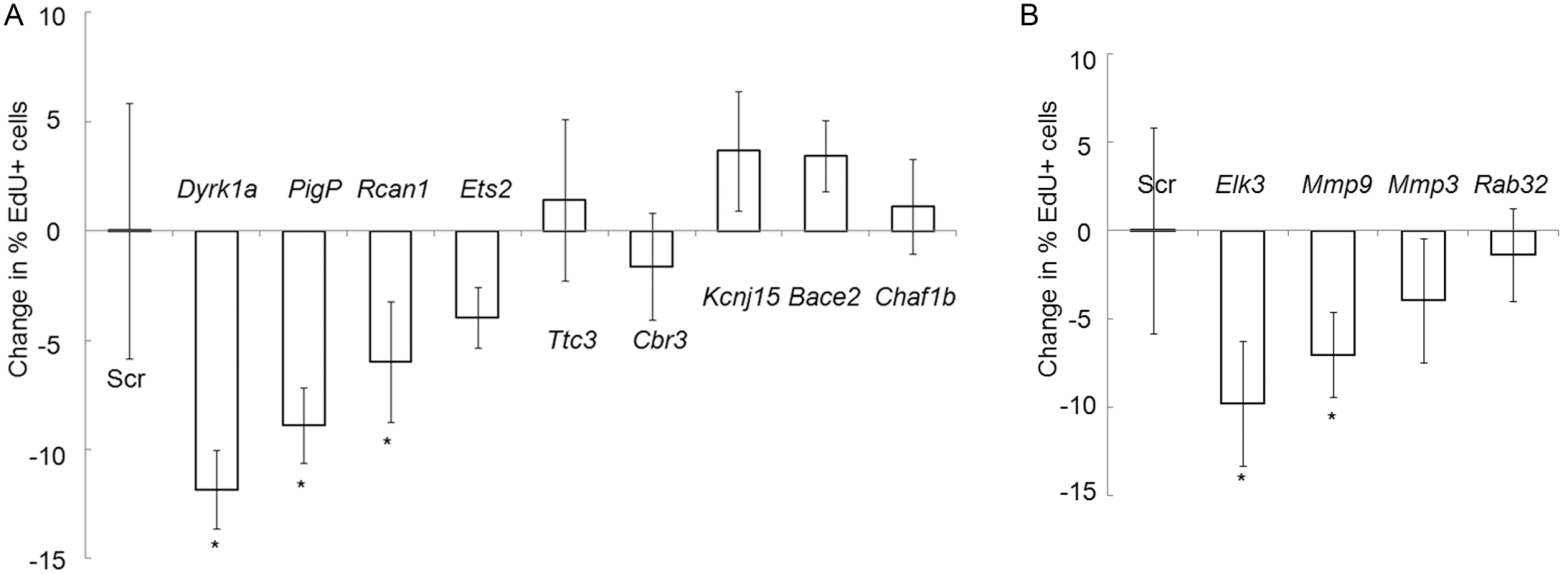
siRNA screening of differentially expressed candidate genes validates effects on TPA-induced proliferation of keratinocytes. The difference in percent EdU positive cells in cultures transfected with the indicated siRNA minus the percent positive after treatment with ScrRNA alone is plotted. (A) The difference in proliferation after TPA when the indicated Hsa21-orthologus gene is targeted. (B) Difference in proliferation with siRNAs directed to disomic candidate genes. * indicates those genes that when knocked down produce significantly decreased or increased TPA-induced proliferation compared to scrambled siRNA (Scr) treated keratinocytes.

Next we considered whether increased Ts keratinocyte proliferation was correlated with increased expression of candidate genes. Transfections with siRNAs targeting the trisomic genes *Ttc3*, *Cbr3*, *Kcnj15*, *Bace2* or *Chaf1b* in Ts keratinocytes did not result in a significant change in the percentage of cells showing EdU incorporation after TPA treatment (Fig 4A). Thus, it appears that none of these genes is necessary for hyperproliferation by itself. However, knockdown of the trisomic genes *PigP*, *Dyrk1a*, or of the Hsa21 ortholog, *Rcan1* (*Rcan1* is on Hsa21 but is not among the trisomic genes in Ts1Rhr) significantly reduced proliferation of TPA-treated Ts keratinocytes, suggesting that up-regulation of these genes is a required part of the TPA proliferation response. *Ets2* knock down had a suggestive but not significant effect on proliferation in Ts cells (Fig 4A).

We then tested four disomic candidate genes that were overexpressed in Ts compared to WT TPA-treated keratinocytes. Knock down of *Rab32* or *Mmp3* did not have a significant effect on proliferation (Fig 4B). However, reduction of either *Mmp9* or *Elk3* transcripts in Ts keratinocytes significantly reduced TPA-induced proliferation.

## Discussion

The genetic mechanisms contributing to malignant transformation are complex and tissue specific. Genetic factors that reduce tumor risk are equally complex and underexplored. The reduction of many tumor types in DS provides one of the best approaches to identify relevant genes and pathways. The observed tumor spectra in individuals with DS and associated mouse models provide a clear example of these principles, as evidenced by an increased incidence of pediatric leukemias [32], the enormous reduction in adult cancers, and the experimentally observed protective effect of trisomy in intestinal adenomas and sarcomas [3, 4]. This study shows another exception to the general observation of reduced solid tumors in DS. We found that a DS mouse model which has reduced incidence for some kinds of solid tumors had a susceptibility to papilloma formation in a chemical carcinogenesis model.

Chemical carcinogenesis in mouse skin is a well-established method to study mechanisms of skin tumorigenesis and to evaluate modifying factors. The tumorigenesis process in this model mimics aspects of human skin carcinoma formation and reflects clearly defined stages of tumor formation in people. Thus the model supports quantification of the effects of modifying factors. The work presented here extends these observations about differential genetic effects, as trisomy for a 32 gene interval orthologous to Hsa21 increased skin tumor multiplicity, contrasting with our previous reports showing that the same regions represses intestinal adenomas [3]. Specifically, our observation indicated that trisomy did not affect initiation as well as malignant transformation, but significantly increased TPA induced growth. This suggests that the trisomic population contained a higher proportion of TPA-responsive cells or that they cycle substantially faster. Our in vitro culture findings mirrored the in vivo proliferative responses of trisomic and euploid skin to TPA.

The discrepant effects of trisomy in various tumor types could reflect tissue specific gene expression, thus we found it useful to specify gene function in defined context. For example, increased dosage of the *Ets2* gene is strongly correlated with protection against intestinal adenomas in *Apc*^*Min*^ mice [3], but is neutral with respect to longevity in the NP-cis cancer model [4]. Here, we found that *Ets2* dosage had a small positive effect on TPA-induced proliferation of Ts1Rhr keratinocytes, a fundamental step in progression of skin cancer. Another example involves *Dyrk1a* and *Rcan1*, both of which are calcineurin pathway regulators. They have been reported to suppress growth of tumor xenografts by inhibiting angiogenesis due to overexpression in host Ts65Dn trisomic mice [7]. In keratinocytes, knocking down *Dyrk1a* expression reduced proliferation. Although we did not perturb Dyrk1a dosage in our in vivo model, it is worth being cautious of its role in tumorigenesis in skin tumors. *Rcan1* is not trisomic in Ts1Rhr mice. Knockdown of *Rcan1* in keratinocytes resulted in reduced keratinocyte proliferation, indicating that it might promote proliferation if overexpressed. Previous studies show that two different *Rcan1* isoforms had opposite effect on tumorigenesis [33, 34], thus we speculate that the oncogenic isoform could be favoured in skin tissue.

Disomic genes are widely dysregulated by trisomy and these effects are evident here [35, 36]. Mutagenesis of *Ras* family protooncogenes, resulting in aberrant upregulation of the *Ras* signalling pathway, is a critical step for formation of skin tumors in the DMBA-TPA model [13]. We found that *Elk3* [37], which is a *Ras*-*Erk* activated transcription factor, was overexpressed in Ts1Rhr keratinocytes following TPA. The *Elk3* gene, knockdown of which produced the largest decrease in TPA proliferation of any disomic gene, is an ETS family transcription factor and, thus, is capable of recognizing the same core consensus DNA binding site as *Ets2*, which is trisomic in Ts1Rhr. Increased expression of *Mmp9*, a gene that is transcriptionally regulated by ETS family transcription factors, has been reported to promote breast cancer progression [38]. We found *Mmp9* to be significantly up-regulated in TPA-treated keratinocytes, 13 fold higher in WT SCC than in normal WT skin and higher still in Ts SCC (Figures A and B in S3 File). Knockdown of *Mmp9* levels significantly reduced the TPA proliferation response in Ts keratinocytes (Fig 4B).

Trisomy is protective against many solid tumor types, thus this unexpected increase in trisomic susceptibility may have implications for clinical care of people with DS. This finding contrasts with previous epidemiological reports from people which describe reduced “skin cancer” incidence or mortality in DS, although the number of cases in these few reports are small and the specific kind of skin cancers is not defined. Here we examine a skin cancer model based on chemical carcinogenesis. While this model has well-characterized parallels with skin cancer progression in people, the initiating events are clearly distinct from the origins of most human skin cancer. The discrepancy between our results and human reports of DS resistance to skin cancer might also derive from the fact that the mouse model we used is trisomic for a relatively small subset of the genes that are trisomic in a person with DS.

Given the multiplicity of different causes for different cancers, it is not terribly surprising that protective effects of trisomy 21 (and mouse models of it) involve diverse mechanisms with greater or lesser effects in different types of cancer. However, genes found to promote or inhibit tumor growth in a “sensitized” DS model might have corresponding effects if alleles exist that are relatively over-expressed in people without DS and it would be useful to screen for such variants. The use of trisomic mouse model to study the genetics of people with DS will further our understanding of the mechanisms that underlie both protection and susceptibility to different forms of this disease.

## Supporting Information

S1 Fig There is no difference in malignant conversion ratio between WT and Ts1 mice. (TIF)

S1 File Characterization of keratinocyte cultures. Immunostaining of keratinocyte with anti keratin-17 (K17) antibody (left) shows the homogeneity of the culture. (Right) Vimentin staining of keratinocyte cultures identifies less than 10% of the cells as fibroblasts (**Figure A**). Flow chart of keratinocyte experiments (**Figure B**).
(TIF)

S2 File Example of cell cycle measured by flow cytometry. Ts keratinocyte cell cycle without TPA treatment (**Figure A**). Ts keratinocyte cell cycle with TPA treatment (**Figure B**). Untreated trisomic and euploid keratinocyte cultures showed no difference in the percentage of cells in S+G2/M phase (**Figure C**). Trisomic keratinocyte cultures had a higher percentage of cells in S+G2/M phase than did euploid cultures after TPA treatment (*p = 0.034, T-test) (**Figure D**). For C and D, 5 independent keratinocyte cultures each from WT and Ts1Rhr were analyzed for DNA content in parallel by flow cytometry.
(TIF)

S3 File Mmp9 expression is elevated in trisomic mouse tissues. *Mmp9* expression level in WT normal skins (n = 6) and SCCs (n = 13). *P<0.001 by T-test (**Figure A**). *Mmp9* expression level in WT SCCs (n = 13) and Ts1Rhr SCCs (n = 14). *P = 0.038 by T-test (**Figure B**). Error bars show standard deviation. All RT-PCR reactions were repeated 3 or more times.
(TIF)

S1 Table List of Taqman probes and SYBR primers for RT-PCR. (TIF)

S2 Table Gene list of siRNA targets from Dharmacon. (TIF)
