# Supplementary figures and images for: Increased Skin Tumor Incidence and Keratinocyte Hyper-Proliferation in a Mouse Model of Down Syndrome
Source: PLoS One. 2016 Jan 11;11(1):e0146570. doi: 10.1371/journal.pone.0146570 (PMC4708994; doi:10.1371/journal.pone.0146570)

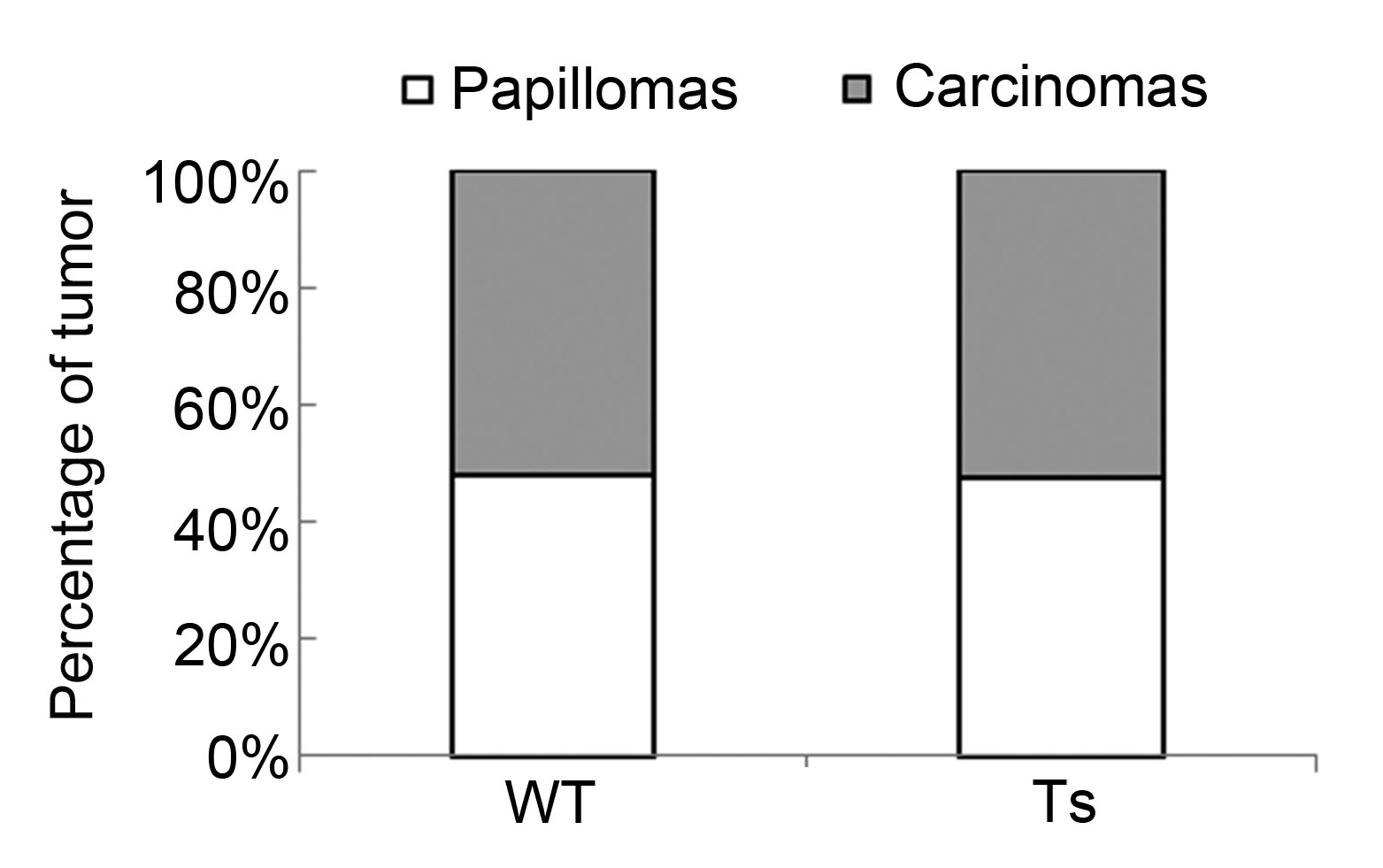

Supplement: S1 Fig — (TIF) [file pone.0146570.s001.tif]

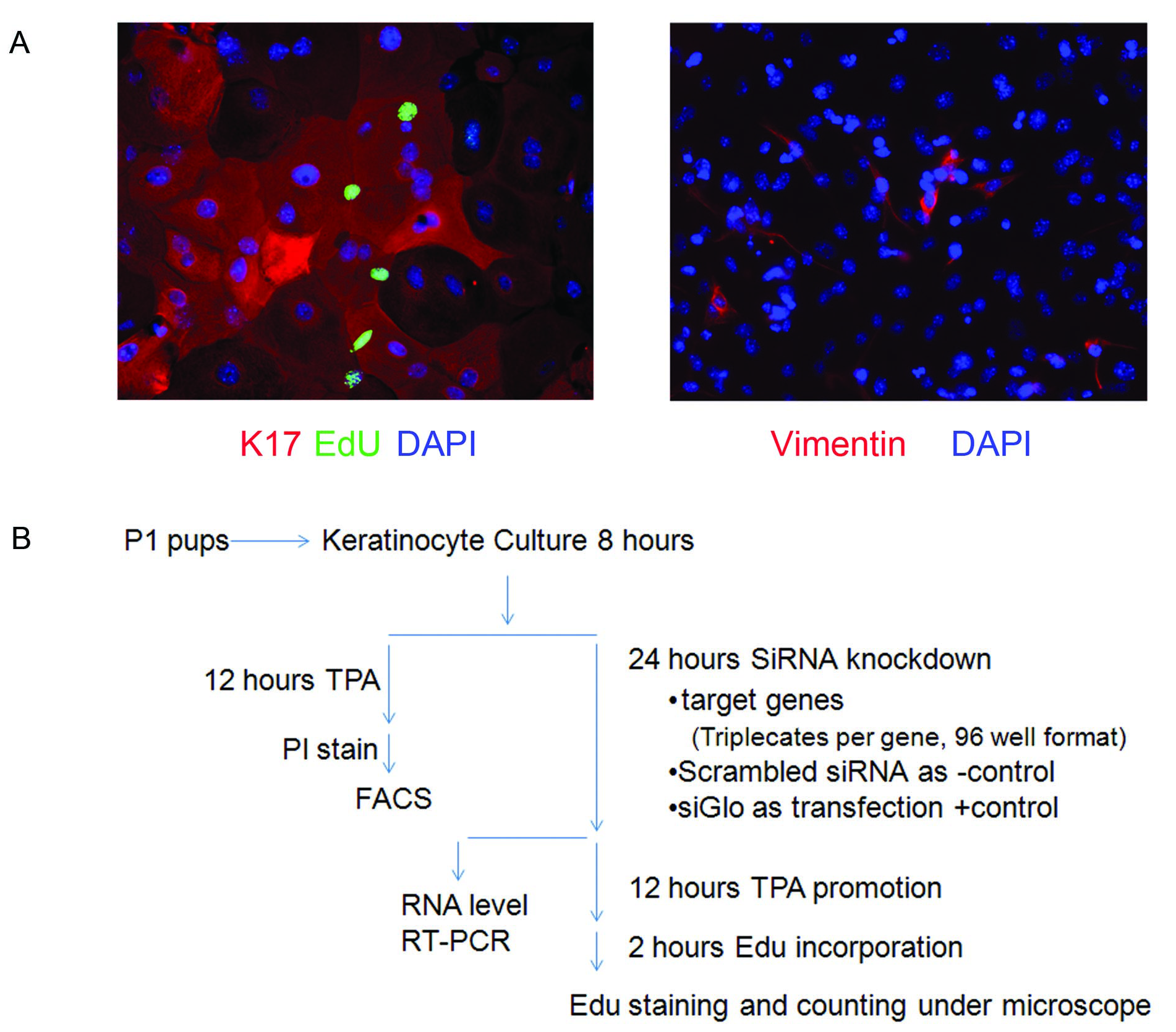

Supplement: S1 File — Immunostaining of keratinocyte with anti keratin-17 (K17) antibody (left) shows the homogeneity of the culture. (Right) Vimentin staining of keratinocyte cultures identifies less than 10% of the cells as fibroblasts (Figure A). Flow chart of keratinocyte experiments (Figure B). (TIF) [file pone.0146570.s002.tif]

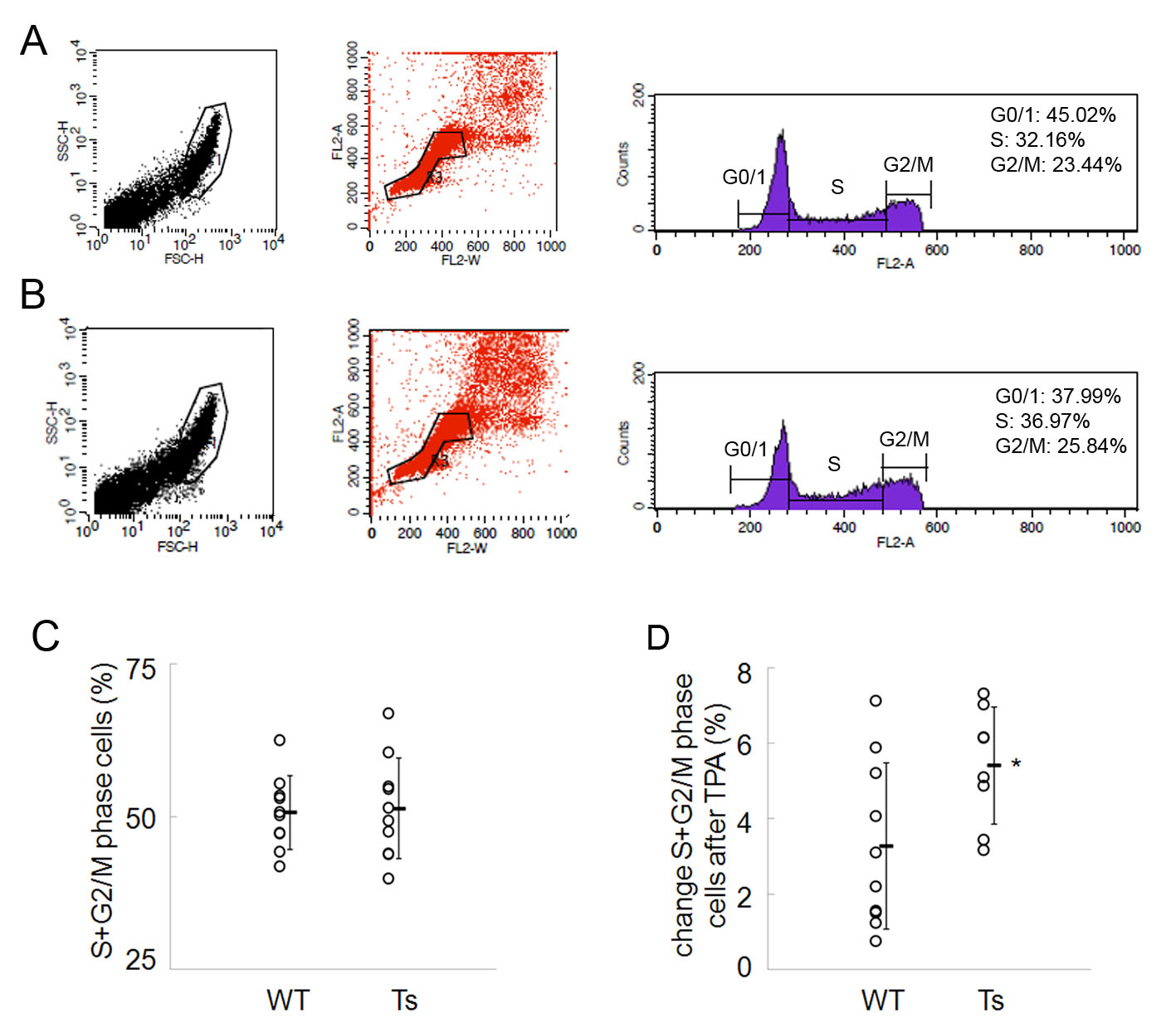

Supplement: S2 File — Ts keratinocyte cell cycle without TPA treatment (Figure A). Ts keratinocyte cell cycle with TPA treatment (Figure B). Untreated trisomic and euploid keratinocyte cultures showed no difference in the percentage of cells in S+G2/M phase (Figure C). Trisomic keratinocyte cultures had a higher percentage of cells in S+G2/M phase than did euploid cultures after TPA treatment (*p = 0.034, T-test) (Figure D). For C and D, 5 independent keratinocyte cultures each from WT and Ts1Rhr were analyzed for DNA content in parallel by flow cytometry. (TIF) [file pone.0146570.s003.tif]

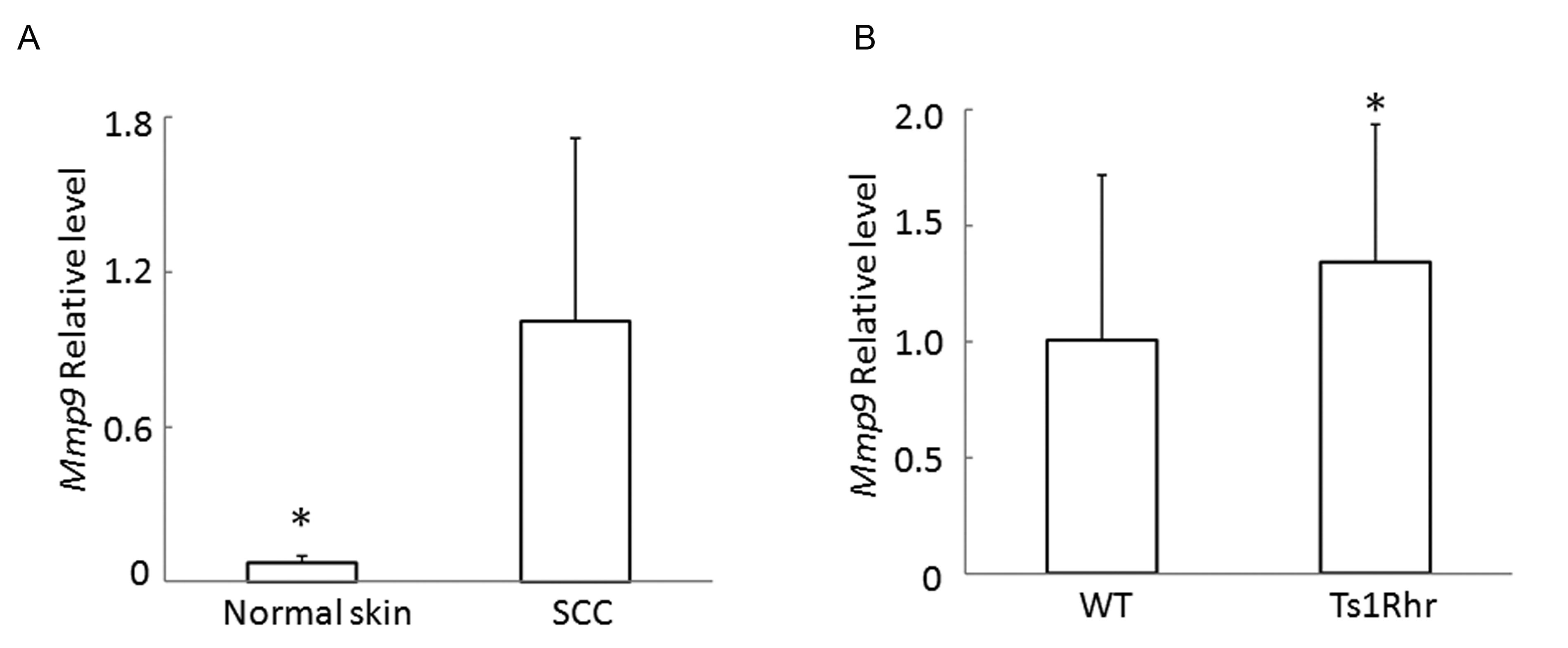

Supplement: S3 File — Mmp9 expression level in WT normal skins (n = 6) and SCCs (n = 13). *P<0.001 by T-test (Figure A). Mmp9 expression level in WT SCCs (n = 13) and Ts1Rhr SCCs (n = 14). *P = 0.038 by T-test (Figure B). Error bars show standard deviation. All RT-PCR reactions were repeated 3 or more times. (TIF) [file pone.0146570.s004.tif]

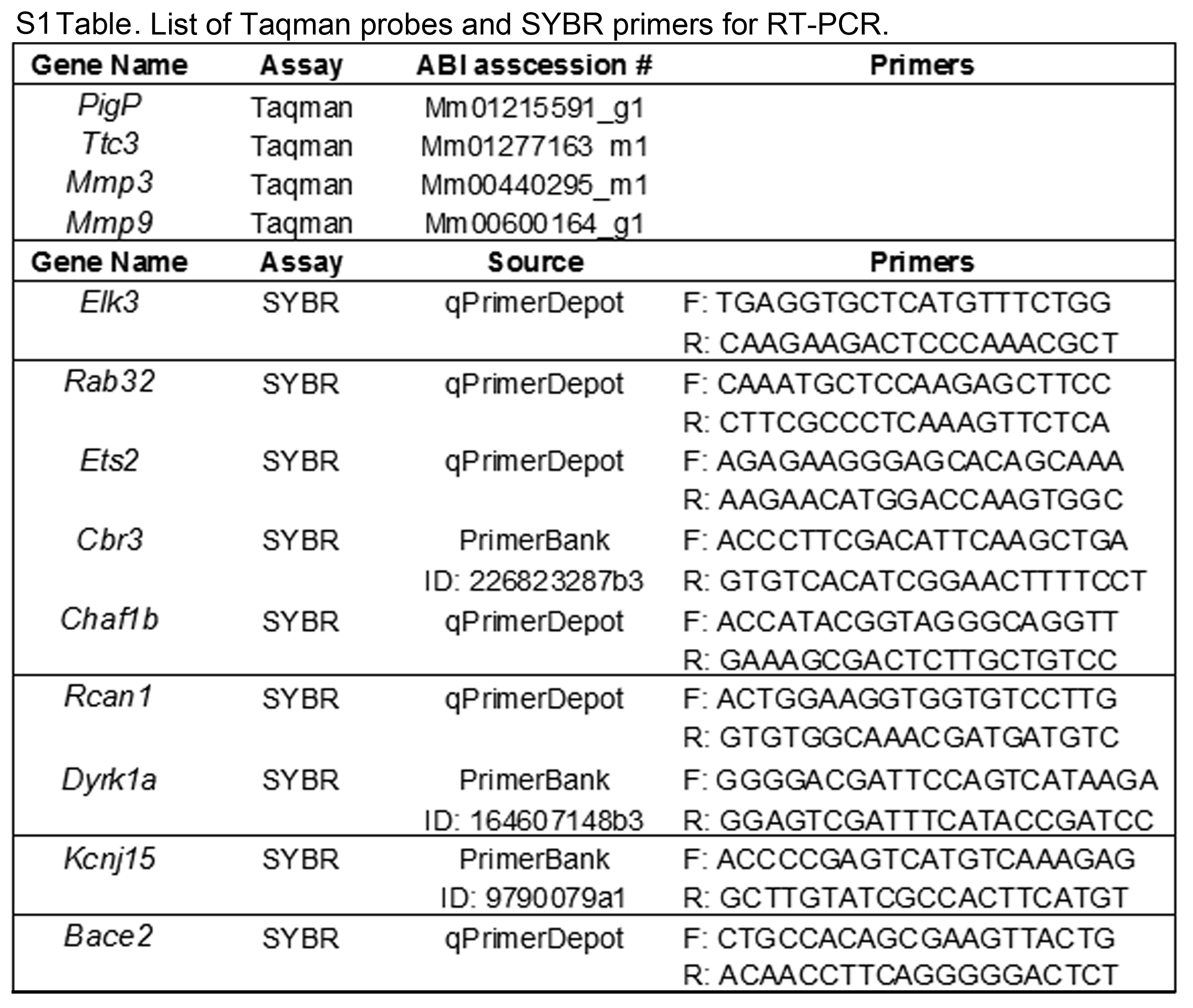

Supplement: S1 Table — (TIF) [file pone.0146570.s005.tif]

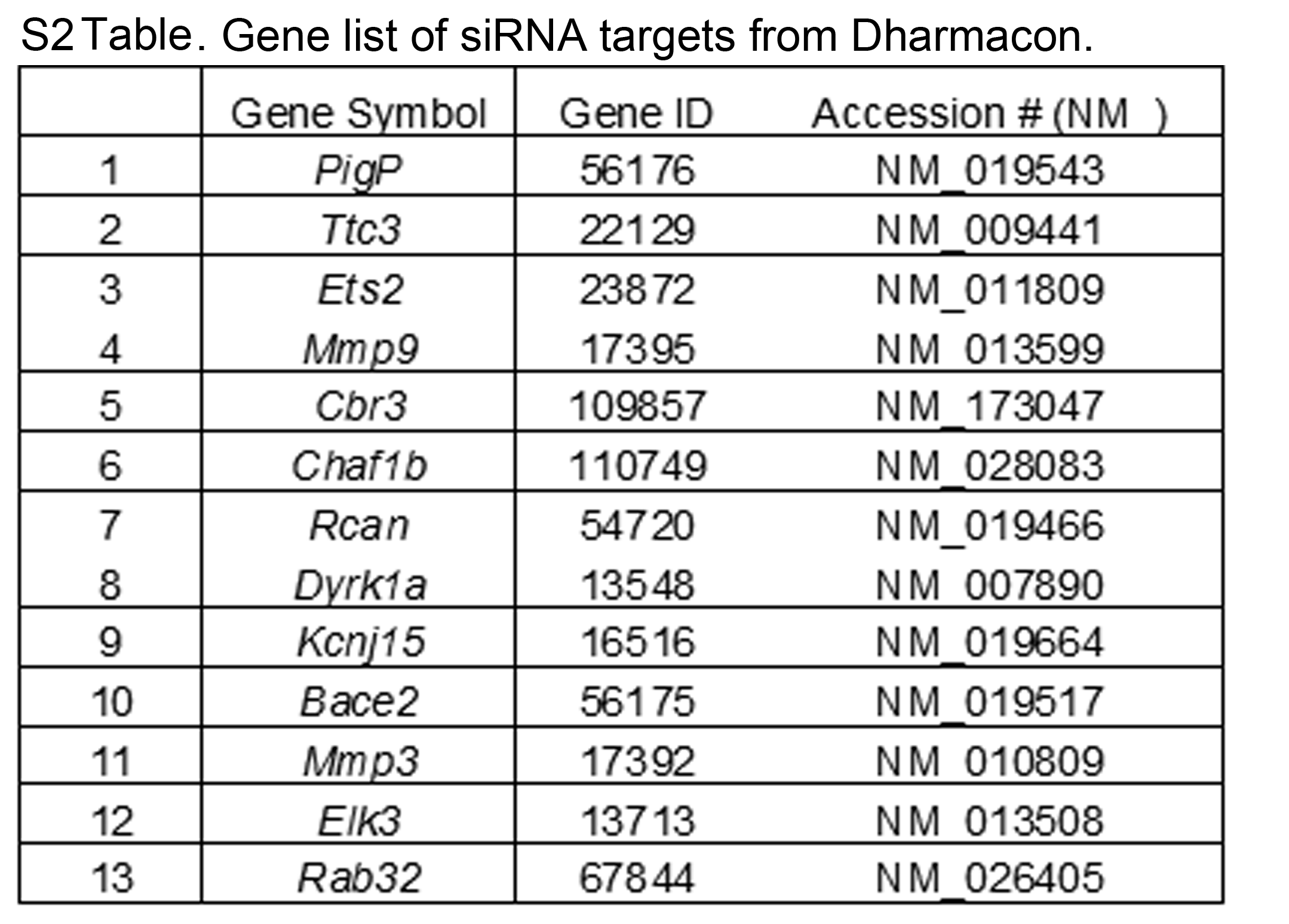

Supplement: S2 Table — (TIF) [file pone.0146570.s006.tif]
